# The Impact of Transcriptomics on the Fight against Tuberculosis: Focus on Biomarkers, BCG Vaccination, and Immunotherapy

**DOI:** 10.1155/2011/192630

**Published:** 2010-12-20

**Authors:** Carlos Rodrigo Zárate-Bladés, Celio Lopes Silva, Geraldo A. Passos

**Affiliations:** ^1^The Centre for Tuberculosis Research, Department of Biochemistry and Immunology, School of Medicine of Ribeirão Preto, University of São Paulo (USP), Ribeirão Preto SP, Brazil; ^2^Molecular Immunogenetics Group, Department of Genetics, Faculty of Medicine of Ribeirão Preto, USP, 14040-900 Ribeirão Preto, SP, Brazil; ^3^Disciplines of Genetics and Molecular Biology, Department of Morphology, Faculty of Dentistry, USP, 14040-904 Ribeirão Preto, SP, Brazil

## Abstract

In 1882 Robert Koch identified *Mycobacterium tuberculosis* as the causative agent of tuberculosis (TB), a disease as ancient as humanity. Although there has been more than 125 years of scientific effort aimed at understanding the disease, serious problems in TB persist that contribute to the estimated 1/3 of the world population infected with this pathogen. Nonetheless, during the first decade of the 21st century, there were new advances in the fight against TB. The development of high-throughput technologies is one of the major contributors to this advance, because it allows for a global vision of the biological phenomenon. This paper analyzes how transcriptomics are supporting the translation of basic research into therapies by resolving three key issues in the fight against TB: (a) the discovery of biomarkers, (b) the explanation of the variability of protection conferred by BCG vaccination, and (c) the development of new immunotherapeutic strategies to treat TB.

## 1. Introduction

The challenge of the World Health Organization Stop TB Strategy (WHO-STOP TB) and the Global Plan to Stop TB [1, 2] is to eradicate tuberculosis (TB) by 2050, while the United Nations Millennium Development Goals aim to halve TB prevalence and deaths by 2015, as compared to their levels in 1990. These are important objectives for public health considering the high burden of the disease, with almost 4 deaths each minute [1]. Moreover, the WHO estimates that 90% of multidrug resistant tuberculosis (MDR-TB) cases are not diagnosed and treated according to international guidelines. Extensively drug-resistant cases of TB (XDR-TB) have been reported in 59 countries since the first description of XDR-TB in 2006 [1].

Since the development of the Bacillus Calmette-Guérin vaccine (BCG) in 1921 and the discovery of the principal anti-TB drugs between 1940 and 1960, essentially no new vaccines or drugs specific for this infection have become available on the market; nonetheless, the use of “-omics” approaches to better understand the host-bacillus interaction, pathogen biology, and host resistance/susceptibility has provided a new impulse for the development of anti-TB strategies. These high-throughput methodologies have recently been applied to delineate the bacillus infective process and to understand the molecular responses of different immune mechanisms. These methods will ultimately lead to a more rational design of novel prophylactic and therapeutic tools against TB.

Microarray technology has been demonstrated to be an especially important tool in these studies and has been applied by different laboratories around the world that are trying to identify specific molecules responsible for key aspects of the disease. Microarray studies have evaluated a myriad of elements of the bacillus and host during the infection. Most studies have focused on identifying novel drug targets and analyzing the gene expression of the host during the course of cell invasion and subsistence [3, 4]. A considerable number of articles have attempted to identify the genetic bases of different grades of virulence among *M. tuberculosis* strains [5, 6], as well as among *M. bovis* [7], that cause TB in bovines and also in humans [8].

A number of studies have evaluated the response of the innate immune cells that the pathogen confronts when it invades the lungs. Special attention has been devoted to macrophages [9] and also to the response of dendritic cells to the infection [10, 11]. These studies underline the importance of these cells as the main orchestrators of the priming of the adaptive immune responses; in TB the Th1 response is essential to effectively clear the infection [12]. These studies were performed with a variety of cell sources (i.e., Different cell lineages, bronchoalveolar cells, and bone marrow-derived cells), microbe strains and culture conditions [9–11, 13]. They indicated that several genes are involved in the cell response to invasion from mycobacteria, and some of them are possible targets for intervention strategies against the bacillus. Although these studies are important for understanding the close interaction between the bacilli and the invaded cells, they produced highly variable results [13]. This makes it difficult to extrapolate the results to the more complex situation in the lungs, where a highly diverse set of cells are present when the mycobacteria arrives and triggers the innate and adaptive arms of the immune response in a multifaceted manner.

Moreover, as will be mentioned later, novel evidence from the immune response during infection, vaccination, and therapy indicates that a complex array of different players takes part in these processes and engages several mechanisms to effectively clear the infection. This means that the immune response functions through the activation of different types of responses that act at various levels to trigger a well-balanced response, rather than the unique activation of the Th1 mediators. This contradicts the previous perception of the immune system that led to the idea that the quantity of the Th1 mediators could reflect the subversion of the pathogen and correlate with the protection by vaccines, and the response to therapeutic regimens [14, 15].

Taking into account the availability of published material on the immune response to TB, this review will focus on the studies that used high-throughput technology, such as microarrays, to examine three currently important issues in the battle against TB: (1) the development of biomarkers for TB and response to treatment, (2) elucidation of BCG vaccine efficacy/failure, and (3) the development of novel therapeutic strategies against the infection by boosting the host immune response.

## 2. Biomarkers of TB

We define biomarkers as molecular features that indicate a defined status of the host in relation to any process or intervention. For TB, biomarkers for different situations are needed. These situations include protection by vaccination, discrimination of latent and active disease to facilitate rapid diagnosis, and treatment outcome or assess relapse risk [16, 17]. Therefore, these biomarkers would be of enormous value to evaluate new candidate vaccines, drugs or any other interventional strategy by accelerating their translation to the clinic [16, 17].

The power of transcriptomics to identify transcriptional biomarkers was previously demonstrated in classical studies on breast cancer, where, through the analysis of the gene expression signatures of primary tumors, identification of a predictive outcome profile was possible [18]. Along these lines, Jacobsen et al. [19] studied the gene expression signature of peripheral blood mononuclear cells (PBMCs) of patients with active TB compared to infected (latent TB) patients and nonsinfected healthy individuals. The evaluation, which combined microarrays, real-time PCRs and a linear discrimination analysis approach, allowed the authors to identify a set of genes including Rab33A, lactoferrin and CD64, that could differentiate between these three groups of individuals. More importantly, the different gene expression patterns of PBMCs allowed for discrimination between individuals with latent TB and patients with active disease and also between patients with recurrent disease and cured TB patients [20]. In this work, the authors used microarray chips to test 50,000 gene sequences, and after discriminatory computational-based analysis, they claimed that they could differentiate the four groups of individuals using a set of 9 genes (RIN3, LY6G6D, TEX264, C14orf2, SOCS3, KIAA2013, ASNA1, ATP5G1, and NOLA3). 

Although no studies have been published, to our knowledge, reporting the clinical validation of these biomarker candidates, the studies mentioned above are important because the rationale for the approach rests on the study of PBMCs as a surrogate for lung tissue. These cells are considered a complex sampling that better resemble the environment at the site of the infection, as opposed to the evaluation of one specific cell type. In our view, this approach is appropriate as a starting point for further studies of the host response during the course of the infection and for distinguishing between good and poor responders that are subjected to interventional strategies (vaccines and therapies).

More recently, Berry et al., [21] reported the analysis of the transcriptome of patients with latent TB and active TB and compared them to healthy subjects. The identification of a 393 gene set enable the authors to identify a gene expression signature that differentiates between latent and active TB. These results were confirmed comparing the transcriptome of patients of different parts of the world demonstrating the power of the strategy adopted. Since this patients were included into the groups analyzed with no other criteria than the clinical data in order to define them as patients with active or latent TB, and that 10%–20% of the patients classified as with latent TB were grouped into the active TB group by their gene expression profiles, the authors suggested that these patients could represent those who will develop active disease in the future [21]. Although this assumption needs to be confirmed by a longitudinal analysis, this study is of outstanding importance not only for giving support to the notion that a transcriptomic approach for TB biomarker development is feasible, but mainly because obtaining such biomarkers seems to be a matter of time.

## 3. Assessment of BCG VaccinationEfficacy/Failure

Another important issue confounding the TB epidemic is why BCG vaccination exhibits high variability in conferring protection among different populations. Although BCG is the most commonly used vaccine in the world with more than two billion doses administered to humans since its development by Albert Calmette and Camile Guérin almost 90 years ago, it only confers good protection against TB in infants and not adults [22]. In several murine models, BCG confers good protection against experimental challenge with *M. tuberculosis* and therefore is commonly used as a “gold standard” for comparing the performance of new vaccine candidates. For this reason, several articles have described the effects of BCG immunization on the immune response in different experimental models and also in humans [23], but the exact immunological mechanisms that leads to BCG protection or to its failure remain poorly understood.

With regard to transcriptomic approaches, the study of Behr et al. [24] is exceptional. These authors evaluated BCG samples used in different parts of the world using DNA microarrays and found a surprisingly high variability between these vaccine samples in specific genome regions associated with virulence. This provided the first explanation for the global divergence in BCG vaccination effectiveness from the microbial perspective; however, this phenomenon might not only be due to bacilli variability. It may also be due to variability in the protection responses among individuals [23]. This has been noted in other vaccination strategies and treatments. 

Understanding BCG failure or efficacy is important because it represents a way to discover the mechanisms underlying protection, which could be useful in the formulation of more efficient vaccines. In this context, host transcriptome analysis following BCG vaccination represents a valid approach due to the robustness of the results and the large set of data obtained. Despite the importance of such studies, only a few have been published describing the transcriptome analysis in the context of BCG vaccination. 

Mollenkopf et al. [25] compared the modulation of gene expression of mice immunized with BCG to those experimentally infected with *M. tuberculosis*. This strategy was useful to identify genes specifically responsive to BCG immunization. A major concern of this evaluation was the use of intravenous inoculation of the vaccine rather than subcutaneous injection, which is used in the classical vaccination protocol of BCG; nevertheless, this study is of great importance because it illustrated a rapid lung transcriptome response after BCG immunization. 

Aranday Cortes and coworkers [26] studied the lung and spleen transcriptome response of vaccinated and un-vaccinated mice. The data analysis allowed identifying expression profiles that could be associated with vaccination efficacy. The mice from this study were intradermally vaccinated and infected with *M. bovis*, and the results confirmed and extended the data from the previous Mollenkopf study [25]. Curiously, so far there have not been any published studies of human samples showing high-throughput transcriptome profiling following BCG immunization. 

Data obtained using a more focused method, such as qRT-PCR, to evaluate 16 immune system genes, showed that groups of children vaccinated with different strains of BCG feature Th1 responses and Treg cells associated genes. Moreover, the results showed that BCG vaccines used in Brazil and Denmark induce a response that is related to the Th1 response in the PBMCs from neonates, while the response to Japan BCG vaccines is related to an inflammatory profile [27]. This study indicates the feasibility of gene expression studies in human samples following BCG vaccination. Moreover, this approach could be useful to better understand the nonspecific, beneficial effects of BCG vaccination in children, a question that remains widely neglected but represents a potential public health problem if BCG is replaced by the new sub-unit vaccines against TB [28, 29].

## 4. Development of Novel Therapeutic Strategies against TB

The emergence of XDR-TB represents a challenge for the development of new, effective drugs with shorter regimens of treatment [1]. The classical antibiotic research and development includes investigation of novel susceptibility pathways of the mycobacterium metabolism that could be used as targets for these compounds. Once again, transcriptome profiling could be useful to identify the differentially expressed genes in mycobacterium strains susceptible or resistant to antibiotics [30].

In parallel with these pharmacological studies, immunostimulation of TB patients has been proposed as an important element that helps improve the outcome of the disease [31, 32]. Candidate approaches are under intensive evaluation as immunotherapeutic options such as *M. vaccae* and a detoxified cellular fragment compost of *M. tuberculosis* named “RUTI”, both tested in clinical trials [33–35]. However, the data indicate the necessity of a more detailed characterization of their mechanisms of action to improve the results observed, in which transcriptomal evaluations could be useful. 

DNA-based vaccines constructed with an *M. leprae* 65 kDa heat-shock protein (DNA-hsp65) and developed by the team of one of us (CLS) represent a new strategy for immune intervention against TB. DNA-hsp65 immunization has been applied successfully in different experimental models of TB. Initially tested as a new vaccine candidate, the data collected since 1992 show that this vaccine stimulates a strong Th1-specific immune response towards the hsp65 immune-dominant antigen [36, 37]. Stimulation with four doses of DNA-hsp65 vaccine can be maintained by using different formulations of microspheres or liposomes or prime-boost strategy reducing up to 16 times the amounts of DNA needed to maintain the protective efficacy [38–40]. Moreover, because mycobacterial Hsp65 is one of the most extensively studied antigens among the several components of the HSP family with regard to their antitumoral properties [41–43], we evaluated the effects of treatment with hsp65 in a phase I clinical trial. Hsp65 intratumoral vaccination resulted in no toxic effects in head and neck squamous cell carcinoma patients [44, 45]. 

In 1999, Lowrie and co-workers [46] showed that the DNA-hsp65 vaccine could be applied effectively to treat active TB, thus becoming the first DNA vaccination protocol to display such properties against an intracellular pathogen. Subsequent studies that demonstrated the advantages of DNA-hsp65 to treat latent TB infection and MDR-TB [47, 48] indicated their importance as one of the most promising molecules against TB [31, 49].

Although significant progress has been made towards a better understanding of the prophylactic mechanism of DNA-based vaccines in driving the immune response, systematic characterization of DNA immunotherapy was not undertaken before the work developed by our research group [50]. We reasoned that the elucidation of the molecular events underlying cellular states that occur during DNA-hsp65 immunotherapy in response to *M. tuberculosis *infection could be evaluated by quantifying differential gene expression using microarray hybridizations and qRT-PCR. 

The results showed that the effects of DNA-hsp65 immunotherapy in mice with active TB could be characterized by gene expression analysis. A high number of transcripts that code for proteins associated with the immune system made it possible to distinguish treated from nontreated animals [50]. Functional analysis of this group of genes suggested that DNA-hsp65 therapy inhibits Th2 cytokines and regulates the intensity of inflammation through fine tuning of the expression of various genes, including those that code for IFN-*γ*, IL-17, lymphotoxin-*α*, TNF-*α*, IL-6, TGF-*β*, iNOS, and Foxp3. In addition, the expression levels of a large number of genes and expressed sequence tags previously unrelated to DNA therapy were modulated. 

A correlation between gene expression and histopathological lesions of the lungs validated the data [50]. Because the effects of DNA therapy were reflected in the modulation of gene expression, the genes identified as differentially expressed could be considered transcriptional biomarkers of DNA-hsp65 immunotherapy against TB.

Thus, we combined the methods that involve characterization of the transcriptional signatures of lungs from mice infected with *M. tuberculosis *and treated with Hsp65 as a genetic vaccine along with microarray, qRT-PCR analysis and finally correlation of the gene expression data with the histopathological analysis of lungs. This was instrumental to obtain a better understanding of the mechanism of DNA-based immunomodulation [50]. Furthermore, we proposed the term “transcriptype” to characterize transcriptome signatures that differ among healthy individuals, infected individuals, or infected individuals treated with DNA immunotherapy [50].

## 5. Systems Biology Tackles the Limitations on the Interpretation of Transcriptional Data

High-throughput transcriptome analysis using microarrays is helping to identify biomarkers for TB, understand BCG efficacy/failure, and develop alternative treatments such as DNA-based immunotherapy. Nevertheless, the relatively few studies in this field reflect the striking difficulty in integrating the large amount of data generated and effectively translating it to clinical use. One of the main reasons for this problem is the lack of integrative tools that provide investigators a more complete and understandable picture of the transcriptome modulation; however, some integrated efforts are being conducted in this area. The Tuberculosis Database (TBDB) at Stanford University and the Broad Institute [51, 52] provide a platform with information regarding the *M. tuberculosis* genome, protein, and gene expression data. They also contain information regarding other mycobacterium-related species, as well as gene expression data from mouse and human samples. Moreover, future expansion of this platform is planned to generate tools that will be able to combine gene expression from the immune system with other data.

Systems biology is a relatively recent discipline that aims to combine, and therefore better understand, the high-throughput sets of genomic, transcriptomic, and proteomic data using bioinformatic tools. Recently, systems biology studies analyzed integrative data to evaluate the effectiveness of the yellow fever vaccine YF-17D [53, 54]. This vaccine represents the most successful prophylactic immunization against an infectious disease due to its high efficacy, safety, and long-lasting protection that can reach 35 years [55]. Even though more than 540 million doses have been administered to humans since its development in 1937, the mechanisms underlying its efficacy remained poorly defined until recently. Seminal studies combined data from human gene expression signatures after YF-17D vaccination using multiparameter flow cytometry analysis for cell type or cytokine evaluation and computational analysis [53, 54]. 

This strategy led to the identification of key elements of the innate and adaptive immune system that predict the immunogenicity and efficacy of YF-17D vaccination, thus providing a new perspective to vaccinology [56] that could be applied to the combat of other infections as TB.

## 6. Concluding Remarks

The application of microarray technology has been demonstrated in different settings. Currently, with the evolution of novel computational analysis tools, the integration and extraction of the vast information generated by this technology are supporting new and exciting discoveries. This systems biology approach is now being applied to the development of novel diagnostics, vaccines and, drugs against TB, as well as to other areas of TB research [57, 58].

In our opinion, some key issues from the “host point of view” that could benefit from these integrative tools are the investigation of BCG vaccine efficacy/failure, the determination of the importance of BCG nonsspecific beneficial effects, and the discovery of TB biomarkers and the evaluation of novel therapeutic strategies, such as immunotherapy and chemotherapy.

## References

[1] WHO Global Tuberculosis Control. A short update to the 2009 report. http://www.who.int/tb/publications/global_report/2009/en/index.html.

[2] http://www.stoptb.org/.

[3] Waddell SJ, Butcher PD (2007). Microarray analysis of whole genome expression of intracellular *Mycobacterium tuberculosis*. *Current Molecular Medicine*.

[4] Haller R, Kennedy M, Arnold N, Rutherford R (2010). The transcriptome of *Mycobacterium tuberculosis*. *Applied Microbiology and Biotechnology*.

[5] Flores-Valdez MA, Morris RP, Laval F, Daffé M, Schoolnik GK (2009). *Mycobacterium tuberculosis* modulates its cell surface via an oligopeptide permease (Opp) transport system. *FASEB Journal*.

[6] Bartek IL, Rutherford R, Gruppo V (2009). The DosR regulon of *M. tuberculosis* and antibacterial tolerance. *Tuberculosis*.

[7] MacHugh DE, Gormley E, Park SDE (2009). Gene expression profiling of the host response to *Mycobacterium bovis* infection in cattle. *Transboundary and Emerging Diseases*.

[8] de Kantor IN, Ambroggi M, Poggi S (2008). Human *Mycobacterium bovis* infection in ten Latin American countries. *Tuberculosis*.

[9] Schnappinger D, Schoolnik GK, Ehrt S (2006). Expression profiling of host pathogen interactions: how *Mycobacterium tuberculosis* and the macrophage adapt to one another. *Microbes and Infection*.

[10] Chaussabel D, Semnani RT, McDowell MA, Sacks D, Sher A, Nutman TB (2003). Unique gene expression profiles of human macrophages and dendritic cells to phylogenetically distinct parasites. *Blood*.

[11] Mortellaro A, Robinson L, Paola RC (2009). Spotlight on mycobacteria and dendritic cells: will novel targets to fight tuberculosis emerge?. *EMBO Molecular Medicine*.

[12] Harding CV, Boom WH (2010). Regulation of antigen presentation by *Mycobacterium tuberculosis*: a role for Toll-like receptors. *Nature Reviews Microbiology*.

[13] Kendall SL, Rison SCG, Movahedzadeh F, Frita R, Stoker NG (2004). What do microarrays really tell us about *M. tuberculosis*?. *Trends in Microbiology*.

[14] Dheda K, Booth H, Huggett JF, Johnson MA, Zumla A, Rook GAW (2005). Lung remodeling in pulmonary tuberculosis. *Journal of Infectious Diseases*.

[15] Rook GAW, Dheda K, Zumla A (2005). Do successful tuberculosis vaccines need to be immunoregulatory rather than merely Th1-boosting?. *Vaccine*.

[16] Wallis RS, Pai M, Menzies D (2010). Biomarkers and diagnostics for tuberculosis: progress, needs, and translation into practice. *The Lancet*.

[17] Parida SK, Kaufmann SHE (2010). The quest for biomarkers in tuberculosis. *Drug Discovery Today*.

[18] Van’t Veer LJ, Dai H, Van de Vijver MJ (2002). Gene expression profiling predicts clinical outcome of breast cancer. *Nature*.

[19] Jacobsen M, Repsilber D, Gutschmidt A (2007). Candidate biomarkers for discrimination between infection and disease caused by *Mycobacterium tuberculosis*. *Journal of Molecular Medicine*.

[20] Mistry R, Cliff JM, Clayfon CL (2007). Gene-expression patterns in whole blood identify subjects at risk for recurrent tuberculosis. *Journal of Infectious Diseases*.

[21] Berry MPR, Graham CM, McNab FW (2010). An interferon-inducible neutrophil-driven blood transcriptional signature in human tuberculosis. *Nature*.

[22] Andersen P, Doherty TM (2005). The success and failure of BCG—implications for a novel tuberculosis vaccine. *Nature Reviews Microbiology*.

[23] Ritz N, Hanekom WA, Robins-Browne R, Britton WJ, Curtis N (2008). Influence of BCG vaccine strain on the immune response and protection against tuberculosis. *FEMS Microbiology Reviews*.

[24] Behr MA, Wilson MA, Gill WP (1999). Comparative genomics of BCG vaccines by whole-genome DNA microarray. *Science*.

[25] Mollenkopf HJ, Hahnke K, Kaufmann SHE (2006). Transcriptional responses in mouse lungs induced by vaccination with *Mycobacterium bovis* BCG and infection with *Mycobacterium tuberculosis*. *Microbes and Infection*.

[26] Cortes EA, Kaveh D, Nunez-Garcia J, Hogarth PJ, Martin Vordermeier H (2010). *Mycobacterium bovis*-BCG vaccination induces specific pulmonary transcriptome biosignatures in mice. *PLoS One*.

[27] Wu BO, Huang C, Garcia L (2007). Unique gene expression profiles in infants vaccinated with different strains of *Mycobacterium bovis* bacille Calmette-Guérin. *Infection and Immunity*.

[28] Roth AE, Stensballe LG, Garly ML, Aaby P (2006). Beneficial non-targeted effects of BCG-Ethical implications for the coming introduction of new TB vaccines. *Tuberculosis*.

[29] Sierra VG (2006). Is a new tuberculosis vaccine necessary and feasible? A Cuban opinion. *Tuberculosis*.

[30] Ma Z, Lienhardt C, McIlleron H, Nunn AJ, Wang X (2010). Global tuberculosis drug development pipeline: the need and the reality. *The Lancet*.

[31] Rook GAW, Lowrie DB, Hernández-Pando R (2007). Immunotherapeutics for tuberculosis in experimental animals: is there a common pathway activated by effective protocols?. *Journal of Infectious Diseases*.

[32] Cardona PJ (2007). New insights on the nature of latent tuberculosis infection and its treatment. *Inflammation and Allergy: Drug Targets*.

[33] Stanford J, Stanford C, Grange J (2004). Immunotherapy with *Mycobacterium vaccae* in the treatment of tuberculosis. *Frontiers in Bioscience*.

[34] Dlugovitzky D, Notario R, Martinel-Lamas D (2010). Immunotherapy with oral, heat-killed, *Mycobacterium vaccae* in patients with moderate to advanced pulmonary tuberculosis. *Immunotherapy*.

[35] Vilaplana C, Montané E, Pinto S (2010). Double-blind, randomized, placebo-controlled Phase I Clinical Trial of the therapeutical antituberculous vaccine RUTI. *Vaccine*.

[36] Silva CL, Palacios A, Colston MJ, Lowrie DB (1992). *Mycobacterium leprae* 65hsp antigen expressed from a retroviral vector in a macrophage cell line is presented to T cells in association with MHC class II in addition to MHC class I. *Microbial Pathogenesis*.

[37] Silva CL, Lowrie DB (1994). A single mycobacterial protein (hsp 65) expressed by a transgenic antigen- presenting cell vaccinates mice against tuberculosis. *Immunology*.

[38] Gonçalves EDC, Bonato VL, da Fonseca DM (2007). Improve protective efficacy of a TB DNA-HSP65 vaccine by BCG priming. *Genetic Vaccines and Therapy*.

[39] Souza PRM, Zárate-Bladés CR, Hori JI (2008). Protective efficacy of different strategies employing *Mycobacterium leprae* heat-shock protein 65 against tuberculosis. *Expert Opinion on Biological Therapy*.

[40] Rosada RS, de la Torre LG, Frantz FG (2008). Protection against tuberculosis by a single intranasal administration of DNA-hsp65 vaccine complexed with cationic liposomes. *BMC Immunology*.

[41] Lukacs KV, Lowrie DB, Stokes RW, Colston MJ (1993). Tumor cells transfected with a bacterial heat-shock gene lose tumorigenicity and induce protection against tumors. *Journal of Experimental Medicine*.

[42] Xu J, Zhu Z, Wu J (2008). Immunization with a recombinant GnRH vaccine conjugated to heat shock protein 65 inhibits tumor growth in orthotopic prostate cancer mouse model. *Cancer Letters*.

[43] Xiangbing HU, Yankai Z, Ming L (2010). The fusion protein of HSP65 with tandem repeats of *β*-hCG acting as a potent tumor vaccine in suppressing hepatocarcinoma. *International Immunopharmacology*.

[44] Michaluart P, Abdallah KA, Lima FD (2008). Phase I trial of DNA-hsp65 immunotherapy for advanced squamous cell carcinoma of the head and neck. *Cancer Gene Therapy*.

[45] Victora GD, Socorro-Silva A, Volsi EC (2009). Immune response to vaccination with DNA-hsp65 in a phase i clinical trial with head and neck cancer patients. *Cancer Gene Therapy*.

[46] Lowrie DB, Tascon RE, Bonato VLD (1999). Therapy of tuberculosis in mice by DNA vaccination. *Nature*.

[47] Lowrie DB, Silva CL (2000). Enhancement of immunocompetence in tuberculosis by DNA vaccination. *Vaccine*.

[48] Silva CL, Bonato VLD, Coelho-Castelo AAM (2005). Immunotherapy with plasmid DNA encoding mycobacterial hsp65 in association with chemotherapy is a more rapid and efficient form of treatment for tuberculosis in mice. *Gene Therapy*.

[49] Lowrie DB (2006). DNA vaccines for therapy of tuberculosis: where are we now?. *Vaccine*.

[50] Zárate-Bladés CR, Bonato VLD, da Silveira ELV (2009). Comprehensive gene expression profiling in lungs of mice infected with *Mycobacterium tuberculosis* following DNAhsp65 immunotherapy. *Journal of Gene Medicine*.

[51] Reddy TBK, Riley R, Wymore F (2009). TB database: an integrated platform for tuberculosis research. *Nucleic Acids Research*.

[52] Galagan JE, Sisk P, Stolte C (2010). TB database 2010: overview and update. *Tuberculosis*.

[53] Gaucher D, Therrien R, Kettaf N (2008). Yellow fever vaccine induces integrated multilineage and polyfunctional immune responses. *Journal of Experimental Medicine*.

[54] Querec TD, Akondy RS, Lee EK (2009). Systems biology approach predicts immunogenicity of the yellow fever vaccine in humans. *Nature Immunology*.

[55] Monath TP (2005). Yellow fever vaccine. *Expert Review of Vaccines*.

[56] Pulendran B (2009). Learning immunology from the yellow fever vaccine: innate immunity to systems vaccinology. *Nature Reviews Immunology*.

[57] Young D, Stark J, Kirschner D (2008). Systems biology of persistent infection: tuberculosis as a case study. *Nature Reviews Microbiology*.

[58] Comas I, Gagneux S (2009). The past and future of tuberculosis research. *PLoS Pathogens*.

